# Ribosomal Protein uL11 as a Regulator of Metabolic Circuits Related to Aging and Cell Cycle

**DOI:** 10.3390/cells9071745

**Published:** 2020-07-21

**Authors:** Mateusz Mołoń, Eliza Molestak, Monika Kula-Maximenko, Przemysław Grela, Marek Tchórzewski

**Affiliations:** 1 Department of Biochemistry and Cell Biology, University of Rzeszów, 35-601 Rzeszów, Poland; 2 Department of Molecular Biology, Maria Curie-Skłodowska University, 20-033 Lublin, Poland; molestak@hektor.umcs.lublin.pl (E.M.); przemek@hektor.umcs.lublin.pl (P.G.); 3 The Franciszek Górski Institute of Plant Physiology, Polish Academy of Sciences, 30-239 Krakow, Poland; m.kula@ifr-pan.edu.pl

**Keywords:** uL11, paralogs, ribosome, translation, aging, lifespan, hypertrophy, cell-cycle

## Abstract

Aging is a biological phenomenon common to all living organisms. It is thought that the rate of aging is influenced by diverse factors, in many cases related to the control of energy metabolism, i.e., the so-called pro-longevity effects of starvation. Translation, regarded as the main energy consumption process, lies at the center of interest, as it has a significant impact on the longevity phenomenon. It has been shown that perturbations in the translational apparatus may lead to a lower rate of aging. Therefore, the main aim of this study was to investigate aging in relation to the protein biosynthesis circuit, taking into account the uL11 ribosomal protein as a vital ribosomal element. To this end, we used set of yeast mutants with deleted single *uL11A* or *uL11B* genes and a double disruptant *uL11AB* mutant. We applied an integrated approach analyzing a broad range of biological parameters of yeast mutant cells, especially the longevity phenomenon, supplemented with biochemical and high throughput transcriptomic and metobolomic approaches. The analysis showed that the longevity phenomenon is not fully related to the commonly considered energy restriction effect, thus the slow-down of translation does not represent the sole source of aging. Additionally, we showed that uL11 can be classified as a moonlighting protein with extra-ribosomal function having cell-cycle regulatory potential.

## 1. Introduction

Aging is a universal biological process that may be generally defined as multifactorial events associated with functional deterioration of physiological and biochemical parameters, strictly dependent on time, which leads to cell death. According to prevailing opinions, aging may occur either as a result of a purposeful program driven by a web of interconnected controlled events or as stochastic, random, and accidental events leading to a metabolic decline [1]. Aging research has been focused on several experimental systems, e.g., yeast [2], nematode [3], fruit fly [4], and mouse or rat models [5], indicating that the energy consumption balance is the main issue. It has been shown that the rate of aging is strongly influenced by diverse factors. For example, in *S. cerevisiae* and *C. elegans*, dietary restriction causes a major lifespan extension [6]. As shown in yeast, the effect of the deficiency in amino acids and perturbations in nutrient signaling pathways may contributes to the pro-longevity effects of starvation [7,8]. Thus, translation, which is an energetically demanding process, is strictly related to modulation of the aging phenomenon: when the protein synthesis declines, the organismal age increases [9]. However, the mechanisms extending the lifespan in connection with alterations in the translation machinery are not yet fully understood. The proposed hypotheses assume that this might be associated with either a decrease in global translation, as shown for the TOR pathway [10] or enhanced translation of specific mRNAs required for lifespan extension, e.g., stress response factors or mitochondrial components [11,12]. Interesting information was provided by analyses involving deletion of genes encoding the translational machinery elements. They showed that deregulation in the translational apparatus itself leads to a lower rate of aging. This includes auxiliary translation factors, such as initiation factors [13], and especially proteins from the large ribosomal subunit [14]; however, not all changes that reduce the rate of protein synthesis extend the lifespan, i.e., depletion of small ribosomal subunits does not have a significant influence on lifespan extension [14]. Thus, ribosomal proteins (r-proteins) appear as one of the longevity regulatory elements. In general, it is regarded that r-proteins are critical for maintaining the integrity of the translational apparatus, contributing to formation and stabilization of the ribosomal particle structure and assisting the ribosome in translation from the qualitative and quantitative point of view [15,16]. Importantly, r-proteins possess an additional functional layer providing regulatory potential for the translation as well as other metabolic circuits [17]. In *S. cerevisiae*, 137 genes code for 79 ribosomal proteins and 59 are encoded by a pair of genes, which are paralogs [18]. It has been proposed that the presence of paralogs is a consequence of genome duplication and is maintained in yeast during evolution [19]. R-protein duplication represents a general phenomenon found also in *S. pombe*, *D. melanogaster*, *C. elegans*, and humans [20], showing that multiplication of r-proteins in the form of paralogs extends the functionality of the translational machinery, especially from the regulatory point of view. However, the role of the duplicated/paralogous r-protein has not been solved, and the regulatory function of the r-protein is a long-standing question. There are several proposals highlighting the regulatory role of r-proteins, including the “specialized ribosome” hypothesis with ribosomal functional heterogeneity in focus [21]; the “depot model” where ribosomes act as reservoirs of regulatory molecules, which are released in response to specific cellular cues [22]; and the “dosage model” referring to ribosomal availability, which is dependent on the level of r-protein expression, explaining the phenotypes associated with the loss of different r-proteins [23]. All these assumptions are not mutually exclusive and show the multi-level functionality of the r-protein, which not only supports the ribosome activity per se, but also modulates versatile metabolic circuits [24]. Thus, r-proteins have been implicated in numerous biological events, beyond translation, with a coined function of extra-ribosomal or moonlighting activity, especially considering their paralogs, underscoring their functional heterogeneity [25]. Thus, as presented above, involvement of r-proteins in the ageing phenomenon may represent one of the moonlighting activities that have never been thoroughly explored in the light of r-protein heterogeneity. As was shown, perturbations in the expression of r-protein isoforms/paralogs may exert diverse effects, in many cases leading to adaptation to stress conditions [26] or even cell death [27]. However, in many cases, the effect of r-protein depletion leads to reduction of the rate of aging, especially in the case of the large 60S subunit proteins [14,28,29,30]. It should be underlined that the research focused on analyses of protein synthesis in relation to aging was focused on the effects of bulk protein synthesis, and thus the molecular cause of aging upon reduction of translation by depletion of r-proteins is in general unknown. Therefore, it has not been resolved whether the extension of the lifespan is directly related to reduction of protein synthesis per se or represents a correlation effect related to the extra-ribosomal activity of r-proteins.

We carried out a wide functional analysis of one of the large ribosomal subunit proteins, uL11 (former name *RPL12*, *L12*) [31], to cast more light on the role of the translational machinery in the aging phenomenon. Protein uL11 is located within the GTPase-associated center (GAC), belongs to the P-stalk structure, and assists the GAC in interactions with translational GTPases (trGTPases) [32]. The uL11 protein represents conserved r-proteins and is regarded as one of the important components of the GAC involved in numerous aspects of the ribosome life cycle, starting from large ribosomal subunit biogenesis, initiation of translation, to elongation. Thus, it contributes to the rate of protein synthesis by stabilizing the so-called productive state of trGTPases [33]; however, in contrast to many r-proteins, especially including those forming the GAC, namely uL10, eL40, or uL6 residing in the GAC [30,34,35], the lack of uL11 is not deleterious for the cell [36]. Recently, it has been shown that uL11 may have regulatory potential, indicating that phosphorylation of uL11 might play a regulatory role in mitosis, implicating uL11 in extra-ribosomal activity [37]. The uL11 protein in yeast is encoded by two paralogous genes, *L12A* and *L12B* [18], named also *uL11A* and *uL11B*, and the role of these two isoforms of uL11 has never been explored. Therefore, the main aim of this study was to investigate the role of the uL11 paralogs, especially using an experimental system based on yeast mutants with depletion of single *uL11A* and *uL11B* genes and double disruptant strain *uL11AB*. We employed the global approach, including biochemical analyses of the translational machinery, cell cycle, intracellular morphology, cell volume, transcriptome analysis, and global chemical composition, together with evaluation of the aging rate.

## 2. Materials and Methods

### 2.1. Strains and Growth Conditions

The following yeast strains were used: wild type strain BY4741 (*MATa; his3 ∆1; leu2∆0; met15∆0; ura3∆0*), isogenic *∆uL11A* (*MATa; RLP12A::HIS3*) and *∆uL11B* (*MATa; RLP12B::URA3*) (Euroscarf), and *∆uL11B (MATa; RLP12B::URA3)* [36]. Yeast cells were grown in a standard liquid YPD medium (1% Difco Yeast Extract, 1% Yeast Bacto-Peptone, and 2% glucose) on a rotary shaker at 150 rpm or on solid YPD medium containing 2% agar. The experiments were carried out at 28 °C.

### 2.2. Kinetics of the Growth Assay

The growth assays were carried out on liquid medium described elsewhere. The yeast cell suspension was incubated for 12 h in a shaking incubator at 28 °C (Heidolph Incubator 1000 at 1200 rpm). The growth was monitored in the Anthos 2010 type 17550 microplate reader at 600 nm by measurements at 2-h intervals for 12 h.

### 2.3. Determination of the Mean Doubling Time 

The mean doubling time was calculated for each analyzed cell as described previously [38]. The doubling time was calculated during the determination of the reproductive potential. The times of the first two reproductive cycles were not taken into account (the first and second doubling times are longer than those of older cells). The data represent mean values from three independent experiments (with 45 cells used in each experiment) with a mean standard deviation (SD). Statistically significant differences were taken at a p of < 0.001 using the one-way ANOVA.

### 2.4. Cell Cycle Analysis

The cells were grown to OD_600_ 0.6–0.7 and harvested by centrifugation. Next, the supernatants were discarded and the pellets were washed with PBS and fixed with cold 70% ethanol for 2 h at −20 °C. The cells were stained with a propidium iodide staining solution (75 μM propidium iodide and 50 Kunitz units per mL of RNAse A in PBS) for 30 min at 37 °C. The samples were analyzed on a flow cytometer LSRII (Becton Dickinson) instrument (excitation 488 nm, emission 575/26 nm), and the cell cycle was determined with FlowJo 7.6.1 software (FlowJo, LLC).

### 2.5. Phenotypic Analysis—a Spot Test for Sensitivity to Congo Red, Calcofluor White, MMS, and Sodium Chloride

The yeast cultures were grown to the exponential phase (OD_600nm_ between 0.8 and 1) and serially diluted to different cellular concentrations as indicated (dilution ratio: 1:10). Five microliters of each cell suspension were spotted onto agar plates containing various concentrations of Congo red (Sigma-Aldrich), calcofluor White (Sigma-Aldrich), methyl methanesulfonate (Sigma- Aldrich), and sodium chloride (Sigma-Aldrich). Growth was registered 48 h after incubation at 30 °C. All phenotypes described in this work were confirmed by multiple tests.

### 2.6. Determination of Translational Fitness by Incorporation of ^35^S-Radiolabeled Methionine 

The cells were grown to OD_600 nm_ 0.5–0.6, washed with deionized water, and resuspended with methionine-depleted SD minimal medium (SD-Met). After 15-min cultivation of the cells at 30 °C, unlabeled-methionine was added to the final concentration of 50 mM and 37 kBq of ^35^S-Methionine (37 TBq/mmol, Hartmann Analytics) was added at time 0 (T0). At 10-min intervals, the OD_600_ of the cultures was measured and 1 mL aliquots of the cultures were collected for protein precipitation with ice-cold 50% TCA (final concentration 10%). Next, proteins collected on Whatman GF/C filters were counted in a scintillation counter Beckman LS6000SE). The translation impairment was determined by comparison of the rate of incorporation (cpm/OD_600_/min) of mutant cells with wild type cells, plotted as a function of time. The results were expressed as the mean percent of wild type activity.

### 2.7. Yeast Polysome Profile Analysis 

Polysome profile analyses were performed by centrifugation of total cell extracts in 7–47% linear sucrose gradients. The cells were grown to OD_600 nm_ (A_600_) 0.4–0.6 in YPD and treated with cycloheximide (CHX) to the final concentration of 100 µg/mL for 20 minutes to stabilize the translating ribosomes on mRNA. Next, the cells were harvested by centrifugation, re-suspended in lysis buffer [10 mM Tris-HCl pH 7.5, 100 mM NaCl, 30 mM MgCl_2_, 100 μg/mL CHX, 1 mM PMSF, 6 mM β-mercaptoethanol, 1 nM pepstatin A, 10 nM leupeptin, 10 ng/mL Aprotinin, 200 ng/mL heparin, and RNase Inhibitor (Sigma-Aldrich)], and disrupted by vigorous shaking with glass beads at 4 °C. The cell lysate was pre-cleared by centrifugation at 12,000 rpm, 10 min, and 4 °C (Rotor 12154-H; SIGMA). Aliquots of the lysate equivalent to A_260_ 12 units were loaded on a linear sucrose gradient, centrifuged for 4.5 h at 26,500 rpm and 4 °C in a SW32Ti rotor (Beckman-Coulter). The resulting fractions were analyzed using an ISCO Brendel Density Gradient Fractionator.

### 2.8. Determination of Budding Lifespan

The budding lifespan of individual mother yeast cells was defined as the number of mitotic cycles (buddings) during cell’s life. After overnight growth, the cells were arrayed on a YPD plate using a micromanipulator. The budding lifespan was determined microscopically by a routine procedure with the use of a micromanipulator as described previously [39]. The number of buds formed by each mother cell reflects its reproductive potential (budding lifespan). In each experiment, 45 single cells were analyzed. The results represent measurements for at least 90 cells analyzed in two independent experiments. The analysis was performed by micromanipulation using the Nikon Eclipse E200 optical microscope with an attached micromanipulator.

### 2.9. Determination of the Total Lifespan

The total lifespan was defined as the length of life of a single mother cell expressed in units of time. The total lifespan was calculated as the sum of reproductive and post-reproductive lifespans. The reproductive lifespan was defined as the length of time between the first and the last budding, and the post-reproductive lifespan as the length of time from the last budding until cell death. The lifespan of the *Saccharomyces cerevisiae* yeast was determined as previously described by Minois et al. [40] with small modification [39]. Ten-microliter aliquots of an overnight grown culture of yeast were collected and transferred on YPD plates with solid medium containing Phloxine B (10 μg/mL). Phloxine B was used to stain dead *Saccharomyces cerevisiae* cells. Dead yeast cells lose membrane integrity and Phloxine B enters the cell space giving pink/red color to the cytosol. In each experiment, 45 single cells were analyzed. During manipulation, the plates were kept at 28 °C for 15 h and at 4 °C during the night. The results represent measurements for at least 90 cells analyzed in two independent experiments. The analysis was performed by micromanipulation using the Nikon Eclipse E200 optical microscope with an attached micromanipulator.

### 2.10. Estimation of the Cell Volume

The cell volume was estimated by optical microscopy and analysis of images collected every fifth cell budding during the routine procedure of determining the reproductive potential. The images were captured with the Nikon Eclipse E200 microscope equipped with the Olympus DP26 digital camera. Cell diameter (d) was measured using the Olympus cellSens Standard software in various planes for each cell, and the mean value was used for calculations. Assuming that each cell has a regular shape similar to the sphere, the cell volume (V) was calculated as V = 4/3π(d/2)^3^. 

### 2.11. Yeast Vacuole Staining

For the vacuole membrane analysis, MDY-64 (Invitrogen; Y7536) was used as described by the manufacturer. The cells were grown to OD_600_ 0.5–0.6 and washed twice with deionized water. Next, the cells were resuspended at 10^6^ cells/mL in 10 mM HEPES buffer pH 7.4 containing 5% glucose. The MDY-64 marker was added to a final concentration of 10 µM and cells were incubated at room temperature for 5 minutes. After harvesting by centrifugation, the supernatants were discarded and the pellets were resuspended in fresh 10 mM HEPES buffer pH 7.4 containing 5% glucose. Fluorescence pictures were taken with Olympus BX-51 microscope equipped with a DP-72 digital camera and cellSens Dimension software.

### 2.12. RNA Sequencing and Analysis of RNA-Seq Data

For the extraction of mRNA, the yeast RiboPure RNA Purification Kit (Invitrogen; AM1926) was used as described by the manufacturer. For mRNA extraction, pelleted 2×10^7^ cells were collected after overnight growth. The quality and yield of the RNA was checked using the TECAN Infinite 200 microplate reader. The samples were stored at a concentration of 300 µg/µL in 5 mg aliquots at −80 °C. The library was prepared using the MGIEasy RNA Library Prep Set (MGI Tech). The libraries were sequenced in DNBSeq technology using the BGISEQ-500 (BGI) sequencer. Library preparation and sequencing was done at BGI (BGI branch in Hong Kong). The data represent the mean values from two independent experiments.

The first stage of the analysis consisted in removal of the adapters using the Cutadapt software (https://cutadapt.readthedocs.io/en/stable). Cutadapt was set up to generate paired-end reads, removing only the 3′ adapters. Based on the adopted parameter, too short reads were removed with 15 as the minimum length threshold. The reads were then mapped with TopHat (https://ccb.jhu.edu/software/tophat/manual.shtml) to the reference *Saccharomyces cerevisiae* S288C genome identified as NCBI 22535 (https://www.ncbi.nlm.nih.gov/genome/15?genome_assembly_id=22535)). The TopHat parameters used were library-type frunstranded no-novel-juncs. Subsequently, the paired-end reads mapped for each gene were counted with the use of HTSeq (https://htseq.readthedocs.io/en/release_0.10.0/index) with no strand-specific differentiation (–stranded=no). The end results were processed in the R free software environment (https://www.r-project.org/) using DESeq2 (https://bioconductor.org/packages/release/bioc/vignettes/ DESeq2/inst/doc/DESeq2.html). Using a BioMart tool (https://www.ensembl.org/ biomart/martview/ 69bf337b5c951e37aa1fbe181375366d), we obtained GO identifiers [41] of the analyzed genes. Using the topGO package (https://bioconductor.org/packages/release /bioc/manuals/topGO/man/topGO.pdf), we assigned GO identifiers with a higher frequency of occurrence among significantly differently expressed genes in the defined comparisons.

### 2.13. Raman Spectroscopy

The Raman spectra of lyophilized yeast were recorded using a Nicolet NXR 9650 FT-Raman Spectrometer equipped with an Nd:YAG laser (1064 nm) and a InGaAs detector. Measurements were performed in the range of 400–1800 cm^−1^ with a laser power of 0.5 W (64 scans per spectrum). Unfocused laser beam with a diameter of approximately 100 μm and a spectral resolution of 8 cm^−1^ was used. Raman spectra were processed by the Omnic/Thermo Scientific software.

To obtain information about the variation of the spectra depending on the type of yeast strains, principal component analysis (PCA) was performed. PCA is a non-parametric method yielding information about similarities and differences between samples. This method was applied for the entire range and individual Raman bands. Statistical and multidimensional analysis was done using PAST 3.0 and Origin 2018 software.

### 2.14. Statistical Analysis

The results represent the mean ± SD values for all cells tested in two independent experiments. The differences between the wild type and the isogenic mutant strains were estimated using one-way ANOVA and Dunnett’s post-hoc tests. The values were considered significant when *p* < 0.05. The statistical analysis was performed using the Statistica 10 software or statistical and multidimensional analysis was done using PAST 3.0 and Origin 2018 software (Raman spectroscopy).

## 3. Results

### 3.1. Lack of A and B isoforms of the uL11 Protein Increases the Doubling Time and Sensitivity to Factors Causing Cell Wall Disorder and Oxidative Stress

**Figure 1:**
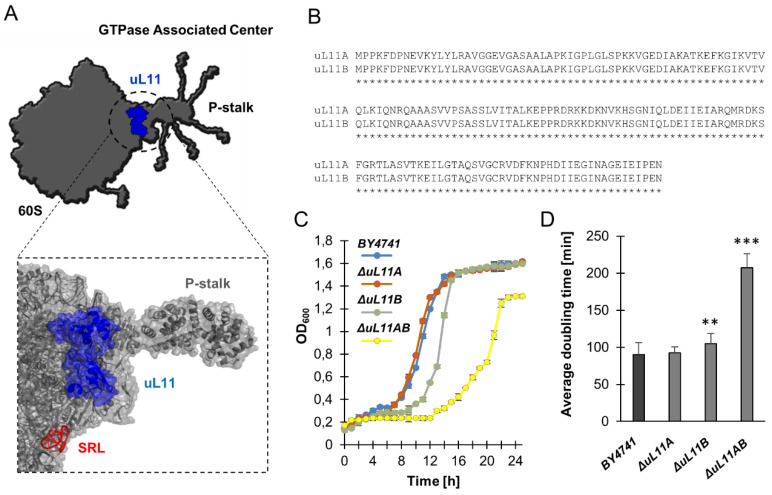
Structural and phenotype overview of yeast ribosomal *∆uL11A* and *∆uL11B* mutants. The model shows the position of the uL11 protein within the GTPase-associated center in the 60S subunit (PDB code 4V6I); rRNA and proteins are indicated as light gray and dark gray colors, respectively. The fitted schematic structure of the uL11A protein is marked in blue. The P-stalk depicts the position of ribosomal P-proteins and uL10 protein (**A**). Amino acid sequence alignment of uL11A and uL11B proteins (**B**). Comparison of growth kinetics on YPD liquid medium (**C**) and average doubling time during reproduction (**D**). Error bars represent standard deviations obtained from three independent experiments. Statistical significance was assessed using ANOVA and Dunnett’s post hoc test (** *p* < 0.01; *** *p* < 0.001) compared to the control.

To obtain insight into the role of the translational machinery in aging, we used *S. cerevisiae* as a model system, and the uL11 ribosomal protein was taken into consideration as an experimental object. The uL11 protein is located in the ribosomal GTPase-Associated Center (GAC) on the 60S ribosomal subunit (Figure 1A). In this study, a set of yeast mutants with deletion of the *uL11* genes was used, including single deletion of A or B paralogs *(∆uL11A* or *∆uL11B)* and the double disruptant mutant *uL11AB (∆uL11AB*). Initially, the growth kinetics was analyzed and the doubling time during the budding lifespan analysis was calculated. 

As shown in Figure 1C, the lack of the *uL11A* gene did not have any influence on growth in comparison to the wild-type strain, while the lack of paralog B exerted a negative effect on the growth; on the other hand, the double disruptant exhibited a slow growth phenotype. Interestingly, deletion of the A and B genes for the uL11 protein increased the average doubling time by substantially more than 100% (Figure 1D), which was manifested as slow growth on the liquid and solid mediums. In turn, despite the identical amino acid sequence in the A and B isoforms (Figure 1B), paralog B had a statistically significantly increased doubling time, confirming the growth rate on the liquid medium. Next, the cell cycle was analyzed. As shown in Table 1, the double disruptant mutant exhibited disorders in the progression from the G1 to G2 phases of the cell cycle with significant accumulation in the S phase. These data complement the result of the doubling time analysis and indicate that the slow-growth effect in the *∆uL11AB* mutant is mainly related to the cell cycle perturbations.

**Figure 2:**
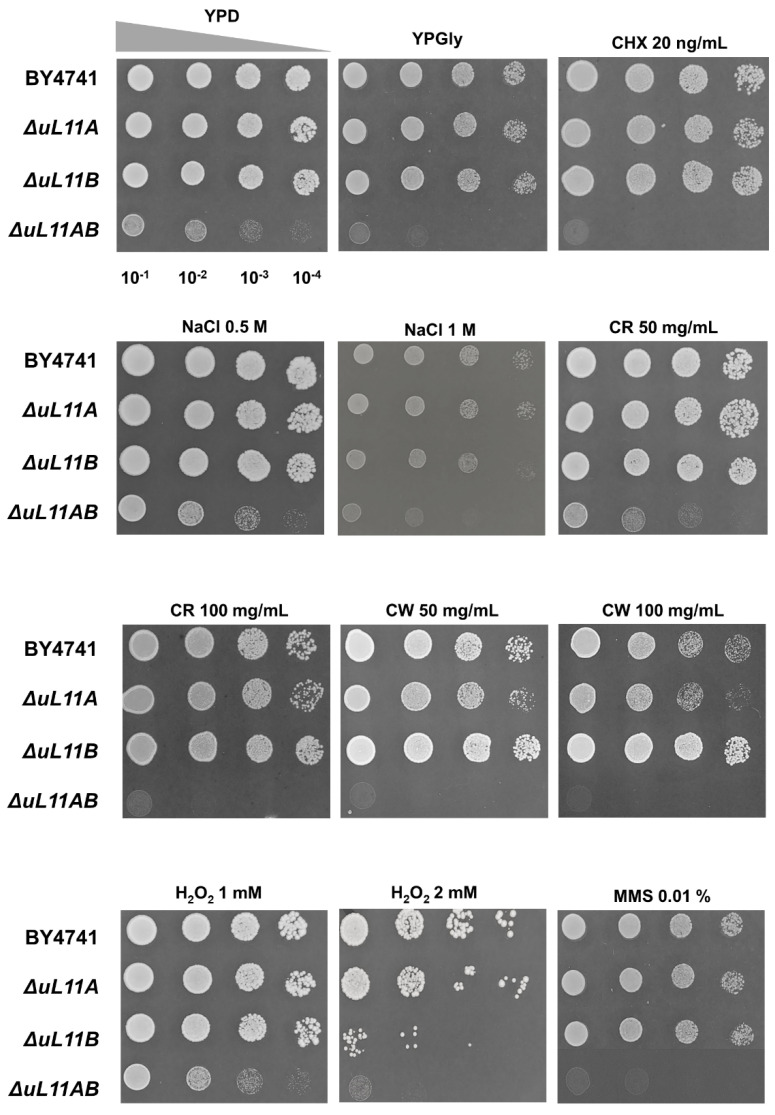
Phenotype screening analysis of yeast mutant strains exposed to different environmental conditions. Yeast strains were grown in YPD medium, spotted onto YPD plates containing the indicated amounts of cycloheximide (CHX), sodium chloride (NaCl), Congo red (CR), calcofluor white (CW), hydrogen peroxide (H_2_O_2_), and methyl methanesulfonate (MMS) and incubated at 28°C. Growth on YPD agar plates was treated as a control. Representative results from two independent experiments are shown.

In the next step, general metabolic analyses were conducted to show the reaction of the mutant strains to various environmental changes. The phenotype screening analysis of the yeast mutants was performed toward various metabolic conditions (Figure 2). The obtained information suggests that the A and B isoforms of the uL11 protein do not have a significant role in adaptation of the yeast cell to the changing environmental conditions. However, the lack of all uL11 isoforms leads to hypersensitivity to factors causing cell wall disorder—Congo red and calcofluor white. Interestingly, the mutants lacking the B isoform or all of them were sensitive to H_2_O_2_, showing that uL11 may have a role in adaptation to oxidative stress. 

### 3.2. Depletion of uL11 Isoforms Negatively Affects Translation

**Figure 3:**
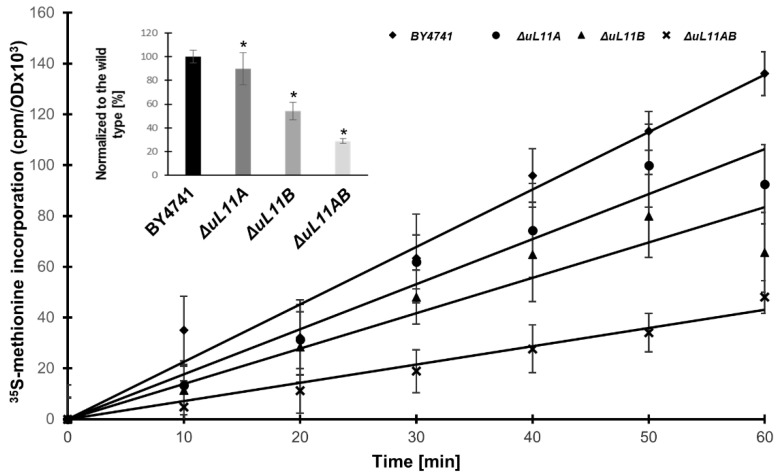
Impact of *uL11* gene deletion on translational efficiency in vivo. Translational fitness determined from the time course of ^35^S-methionine incorporation. The inset shows the translational efficiency normalized to the wild type strain. Error bars represent standard deviations obtained from three independent experiments. Statistical significance was assessed using Student’s test (* *p* < 0.01).

The deletion of ribosomal proteins of the large ribosome subunit often affects the rate of translation [14]; therefore, we subsequently examined the global translational rate by measurement of radiolabeled ^35^S-methionine incorporation in the single and double disruptant mutants, compared to the wild type strain, i.e., the so-called translational fitness. The translation efficiency varied, depending on the strain. The lack of all uL11 isoforms reduced the translation efficiency by 70% compared to the wild strain, but, in the case of single deletions, the lack of the A or B forms reduced translation by approximately 10% and 40%, respectively (Figure 3). 

**Figure 4:**
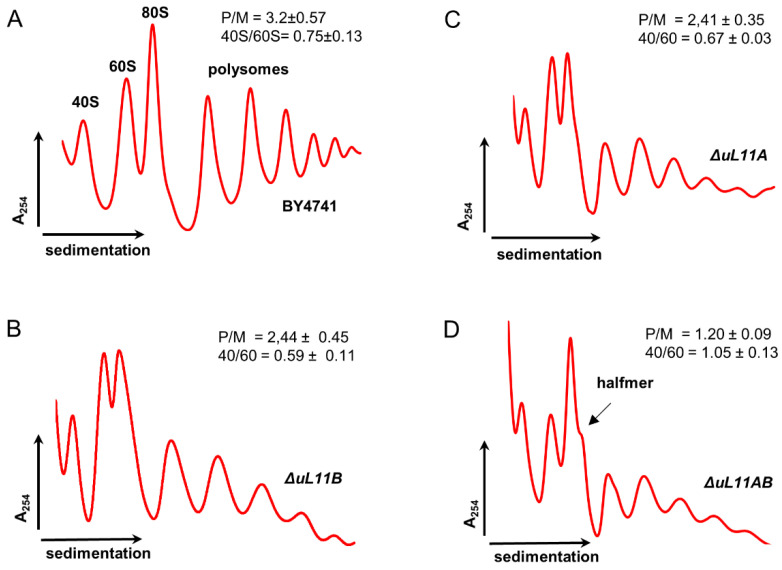
Polysome profile analysis. Polysome profiles obtained for the control BY4741, *∆uL11A*, *∆uL11B*, and double disruptant *∆uL11AB* strains. The sedimentation vector of the ribosomal fractions is indicated by a horizontal arrow, and optical density analysis at 254 nm is shown on the y-axis; the position of individual ribosomal subunits and half-mers is indicated. (**A**) wild type cells; (**B**,**C**) the single deletion strains; (**D**) double disruptant mutant. Insets—P/M, the polysome to monosome ration; 40S/60S—ratio of small to large ribosomal subunits.

Importantly, the recorded differences in the rate of global translation efficiency observed for all deletion mutants corroborate our previous analyses, showing a correlation between growth defect and translational perturbations. The double disruptant strain showed a low translational rate, which resulted in the slow growth phenotype as well as perturbations in cell cycle progression and oxidative stress sensitivity. Interestingly, the lack of the B isoform also exerted a strong effect on the protein synthesis rate, which was also reflected in growth perturbation. Next, we analyzed the performance of the translational machinery by examination of individual ribosomal elements in the translational process using the polysome profile analysis. The single mutants exhibited altered polysome profiles in comparison to the wild type cells; especially the P/M parameter indicated that the polysomal fraction is decreased, displaying perturbations in the translationally active fraction of ribosomes (Figure 4). In turn, the lack of both A and B isoforms resulted in dramatic changes in the polysome profile. Particularly important is the emergence of the so-called half-mers, well depicted on 80S and marked on polysomal fractions (Figure 4, arrow). The half-mers represent the 43S pre-initiation complex on the mRNA and may arise through perturbations in 60S biogenesis or a defect in the initiation step during the translational cycle. 

Additionally, the 40/60 ratio changed, showing accumulation of the 40S subunit and deficiency of 60S, indicating that half-mers probably arise because of the 60S biogenesis defect. Similarly, the double disruptant mutant had a strongly reduced P/M ratio, suggesting that the translationally active machinery was perturbed, supporting at the same time the very low translational fitness and explaining the slow growth phenotype. 

### 3.3. The Lifespan is Affected by the Lack of the uL11 Protein

**Figure 5:**
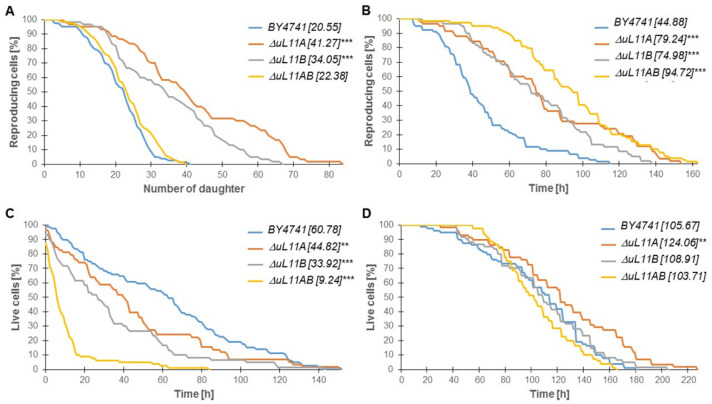
Aging analysis of uL11 yeast mutants. Comparison of the budding lifespan (**A**), reproductive lifespan (**B**), post-reproductive lifespan (**C**), and total lifespan (**D**) of the haploid wild type yeast strain BY4741, isogenic single mutants *∆uL11A*, *∆uL11B*, and double mutant *∆uL11AB* after cultivation on solid YPD media. Statistical significances were assessed using ANOVA and Dunnett’s post hoc test (** *p* < 0.05, *** *p* < 0.001). Data represent mean values from two independent experiments. The mean values for 90 total cells from two independent experiments are shown in parentheses.

Having determined that the depletion of all uL11 isoforms in the yeast cells reduced the translation rate significantly, we asked whether it affected the aging, considering several aspects, such as the budding lifespan and longevity, measured at the single cell level. The so-called budding lifespan is a measure of the number of daughter cells produced by the yeast mother cell. It is part of a standard approach to determine the cell age (so-called replicative lifespan), but it actually determines its fertility rather than aging. The cell longevity or survival rate is determined by analysis of the total lifespan; it shows the cell lifetime expressed in units of time (as in the case of other organisms) and is a sum of the reproductive lifespan and the post-reproductive time (from the last budding to death). As shown in Figure 5A, the lack of one of the *uL11A* or *uL11B* genes led to a statistically significant increase in the mean budding lifespan (*p* < 0.001), with up to 41 and 34 daughter cell produced by the single deletion strains, respectively, vs. 20 in the wild type strain. In turn, in the mutant strain with both genes deleted, there is no significant increase in the budding lifespan. On the other hand, in the case of the double disruptant mutant, a significant increase in the average doubling time was recorded, but it was not correlated with production of daughter cells (Figure 5A,B).

All analyzed strains had a statistically significantly shortened post-reproductive time (Figure 5C), and an especially interesting observation was made for the double disruptant strain, which had a drastically reduced post-reproduction time down to mean 9 h vs. 61 h in the wild type strain. Finally, the analysis of the total lifespan (the measure of cell longevity) yielded an interesting finding, showing that the deletion of *uL11A* led to a statistically significant increase in life expectancy (Figure 5D), while the other strains displayed a similar value to the wild type strain. Thus, it can be concluded that mild deregulation of the protein synthesis rate increases the longevity, while more pronounced changes bring a rather deleterious effect. 

### 3.4. Changes in the Cell Volume During the Budding Lifespan

**Figure 6:**
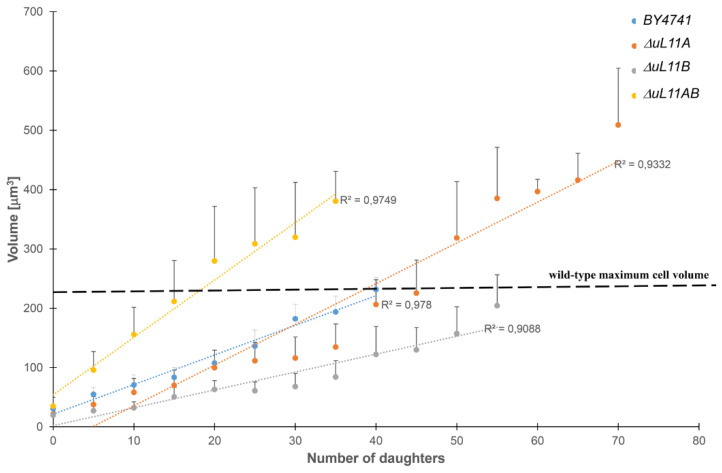
Dependence of the cell volume on the number of daughter cells produced by mother yeast cells. Changes in the cell size during the yeast reproductive lifespan were estimated by analysis of microscopic images recorded every fifth cell cycle during the determination of the budding lifespan. The results represent values for all cells tested in two independent experiments (90 cells). The bars indicate standard deviations.

Since the mutant strains displayed different budding lifespan and longevity parameters, we analyzed the kinetics of cell volume changes during the subsequent steps of doublings events. Importantly, the analysis of the volume achieved by the yeast cells is particularly important in the light of the aging-related hypertrophy hypothesis, which provides an alternative explanation for the replicative aging of yeast [42]. It assumes that the cell loses the ability to produce daughter cells (make subsequent doublings) when it reaches its maximum volume. The use of the yeast mutant lacking one or two particular uL11 proteins provided striking information; the cell volume of the strain lacking uL11A protein, which was able to produce up to 41 daughter cells (Figure 5A), exceeded the volume of the wild type. On the other hand, the strain lacking uL11B, also producing a higher number of daughter cells, had a smaller volume vs. the wild type strain (Figure 6). Striking findings were provided by the analyses with the double disruptant mutant; the growth volume per doubling is extraordinary high and the value exceeds significantly the maximum volume that the wild type strain may achieve. These results make an important contribution to the understanding of the aging phenomenon, suggesting that the hypertrophy hypothesis as a factor regulating the proliferation potential does not correlate with the cell volume.

### 3.5. Deletion of uL11 Genes Affects Vacuole Biogenesis

**Figure 7:**
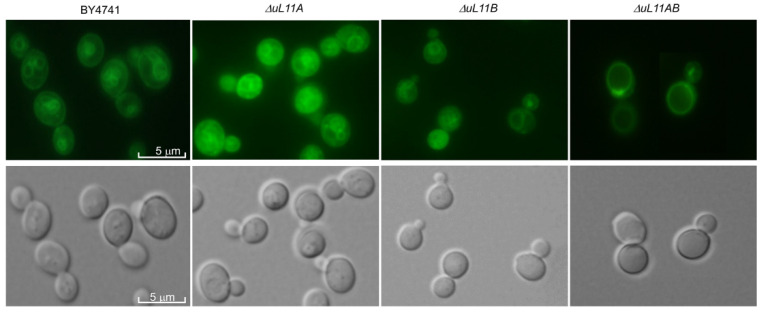
Vacuole morphology analysis. Cells were grown on YPD to an early log phase and stained using a vacuolar marker. Fluorescence pictures were taken with an Olympus BX-51 microscope equipped with a DP-72 digital camera and cellSens Dimension software. Representative results from two independent experiments are shown (1000× magnification).

Changes in the cell volume can be associated with many alterations in cell metabolism, including cell wall biosynthesis and vacuole biogenesis [43]. Therefore, we also focused our attention on the vacuole, a vital yeast element determining cell metabolism. The vacuoles were stained to see whether their morphology and numbers did not change, as the changes usually reflect problems in their biosynthesis. Importantly, as shown in Figure 7, removal of both uL11 protein isoforms significantly affected the vacuole morphology, showing formation of one large integrated structure, indicating unusual fusion of all vacuoles. On the other hand, the wild type strain and single mutants display a similar pattern of vacuole morphology, with several small vacuoles (Figure 7). The observed phenomenon of vacuole abnormalities in the double disruptant mutant along with cell wall dysfunction (shown in Figure 2) may explain its behavior, as the strain showed cell rupture immediately after accomplishing reproduction (data not shown). 

### 3.6. Transcriptional Changes are Stimulated by uL11 Deletion

**Figure 8:**
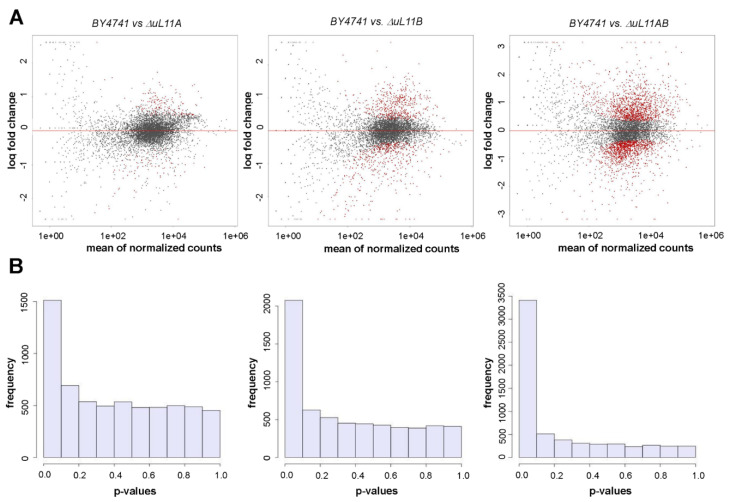
Gene expression analysis. Relationship between logarithmic fold change and normalized averaged expression for a given gene (each point on the graph represents the value for the single genes). Negative values mean that the expression in the mutant was lower than in the BY4741, while positive values mean that the expression in the BY4741 was lower than in the mutant. Genes with FDR < 0.05 are marked in red (**A**). Histogram of *p*-values obtained in a statistical test (Wald test) for the significance of the change in gene expression prepared with DESeq2 software (**B**).

To evaluate cell responses to the *uL11* gene deletion, we performed transcriptome analysis in *∆uL11A* or *∆uL11B* and *∆uL11AB* mutants in comparison to the isogenic BY4741 wild type strain. The total RNA was isolated from the wild type strain and the mutants and prepared libraries were sequenced by the next-generation sequencing approach. Initially, the relationship between the logarithmic fold change and the normalized and averaged expression for a given gene was analyzed. As shown in Figure 8A, the *∆uL11A* mutant had the smallest number of significantly up- and downregulated genes compared to the wild type strain. The intermediate parameters were found for the strain lacking the uL11B protein; in turn, the removal of both isoforms significantly affected the expression of large groups of genes, having negative and positive effects. The results are confirmed by the numerical analyses presented in Figure 8B, which showed global changes considering fluctuations in all genes expressed as frequency. Once again, in the case of the *∆uL11A* mutant, approximately 1500 genes had perturbed gene expression compared to the wild type strain; the lack of the uL11B protein exerted a more pronounced effect, with a change in the expression of over 2000 genes. The most pronounced effect was recorded for the double disruptant strain having the largest number of differentially expressed genes (3500) (Figure 8B). 

Next, we selected Gene Ontology (GO) identifiers for the cellular component category. Fisher’s *p*-values were determined for this category (using the topGO package). According to Fisher’s ranking for the double disruptant mutant, which showed the most significant gene expression fluctuations, the changes were noted in the following metabolic events: nucleolus, pre-ribosome, microtubule, cytoskeleton, spindle microtubule, organelle lumen, vacuole, and fungal-type cell wall (Table S1). In the case of the *∆uL11A* mutant, significant changes according to Fisher’s ranking were reflected in the lower number of metabolic events, such as cytosolic ribosome, ribosomal subunit, cytosolic part, cytosolic large subunit, ribosomal subunit, pre-ribosome, small ribosomal subunit, nucleolus, and fungal-type cell wall (Table S1). In the case of the *∆uL11B* mutant, according to Fisher’s ranking, the significant changes resembled those for *∆uL11A* and for the double disruptant mutant (Table S1).

### 3.7. Cross-Differentially Expressed Genes (DEGs) Reveal Core Biological Processes and Pathways in Response to uL11 Deletion 

**Figure 9:**
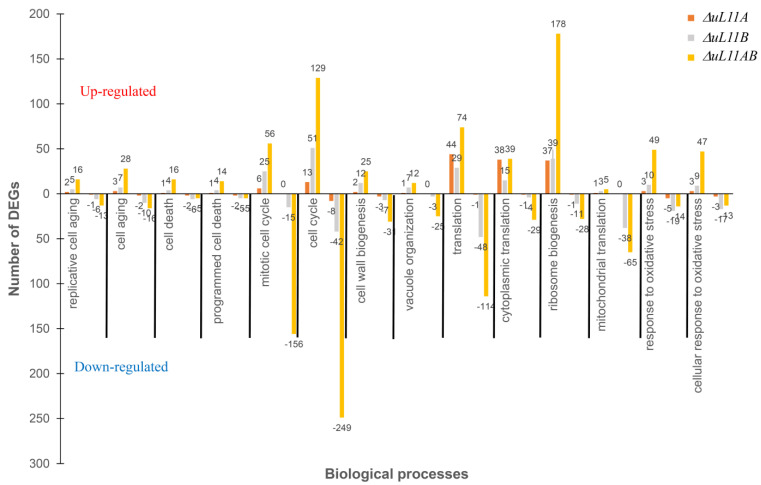
Gene Ontology (GO) enrichment of DEGs. GO enrichment of up- and downregulated DEGs were performed using biological process classification. The GO term was considered enriched significantly when the *p* values of Fisher’s exact test were < 0.05.

Next, we focused on assessing the number of DEGs in several categories of biological processes represented predominantly in the GO analysis. We analyzed the main pathway related to aging, cell death, cell cycle, translation, ribosome, and cell wall biogenesis, also including stress. As shown in Figure 9, the dominant effect was mainly noted in the area of translation including ribosome biogenesis and in the cell cycle and response to the oxidative stress. The most pronounced effect was associated with deletion of both uL11 paralogs. Depletion of A and B forms exerted the strongest effect on the translational apparatus, leading to overexpression of 291 genes related to translation and ribosome biogenesis GO groups and downregulation of 171 genes in the same GO sections. 

The most striking observation is related to the cell cycle pathway, which belongs to the second GO group affected by the lack of all uL11 protein isoform; 185 genes were upregulated and 408 genes were downregulated, making this pathway the most negatively affected metabolic event. Additionally, the expression of genes related to oxidative stress, aging, cell wall biogenesis, and vacuole organization are positively affected; thus, apart from perturbations in translation related genes and cell cycle, the double disruptant strain displayed stress profile expression of genes involved in response to various stress conditions. Considering deregulation in the number of DEGs in connection with the single deletion mutants, it should be pointed out that lack of uL11A or -B proteins resembles the transcriptomic pattern found for the double disruptant mutant but with significantly different intensity (Figure 9). The smallest changes were recorded for the mutant lacking uL11A; however, regarding translation (upregulated genes), the fluctuations were comparable to the mutant lacking uL11B. On the other hand, the depletion of uL11B exerted a stronger effect compering to uL11A mutant, with the transcriptome pattern resembling that found in the double disruptant mutant but with lower intensity. 

### 3.8. Deletion of uL11 Genes Significantly Changes the Chemical Composition in Yeast

**Figure 10:**
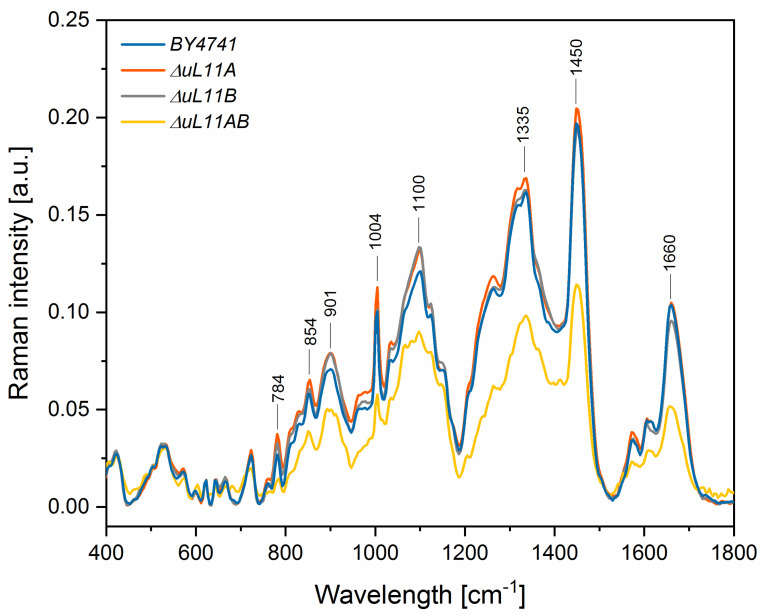
Raman spectra of the yeast cultures with regions corresponding to vibrations of functional groups building RNA, polysaccharides, phospholipids, nucleic acid, carbohydrates, proteins, and lipids.

The phenotype profiling and transcriptomic analysis showed that depletion of uL11A or -B, and especially all isoforms, led to cellular dysfunctions, especially in the case of translation apparatus and cell cycle metabolism, with smaller effect on stress response metabolism. To gain more insight into the chemical composition of the yeast mutant strains, we applied the Raman spectroscopy. All analyzed samples showed spectra with numerous peaks reflecting cellular chemical composition; the pattern of the peaks was similar in all mutants but had different intensities. In the Raman spectrum, several distinct peaks were recognized, which correspond to vibrations of functional groups in RNA (784 cm^−1^) and other nucleic acid (1100 cm^−1^), amino acids (901 and 1004 cm^−1^), polysaccharides (854 cm^−1^), phospholipids (854 cm^−1^), proteins (1335 cm^−1^), lipids (1450 cm^−1^), and amide I (1660 cm^−1^) (Figure 10). The intensities of well-resolved peaks and their statistical comparison to the control group (BY4741) are presented in Table 2. The differences in the peak intensities result from the various amounts of chemical compounds in the studied yeast cells, indicating variations in the chemical composition in the analyzed yeast mutants vs. the wild type strain; thus, the analysis can be regarded as a metabolomic approach. The analysis of the double disruptant strain showed that all distinct signals in the Raman spectrum displayed statistically significantly lower peak intensities in comparison to the control yeast, indicating that the metabolic profile in the mutant strain had significant alteration. 

**Figure 11:**
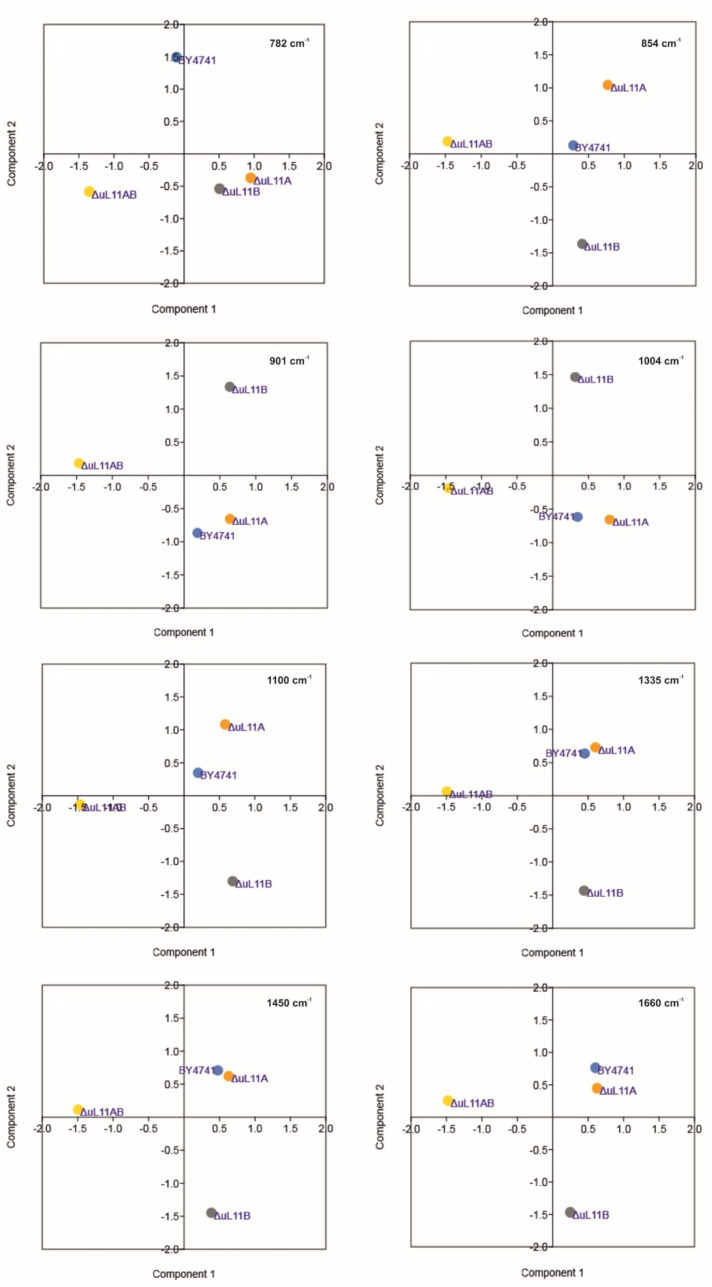
Principal component analysis (PCA) of yeast data from Raman spectroscopy. Two-dimensional (2D) score plot of yeast mutants presented through measurements of spectral functional groups showing differences in the metabolic compositions. For PCA analysis, the intensity of Raman peaks was used at the appropriate wavelength for the functional group for eight independent replicates for each object. Insets on the top right represents the individual peak values taken for analysis, recorded by Raman spectroscopy analysis.

On the other hand, the single deletion mutants, especially the one lacking the uL11A protein, were characterized by similar or significantly higher content of chemical compounds, in comparison to the BY4741 yeast. Especially, the intensities of peaks corresponding to the vibrations from the stretching (O–P–O) RNA (784 cm^−1^) and symmetric benzene/pyrrole in-phase and out-of-phase breathing mode of tryptophan and phenylalanine (901 cm^−1^) were significantly higher for paralog A and partially for B, in comparison to the control group derived from the wild type. Additionally, the strain lacking the A paralog showed significantly higher peaks originating from vibrations of polysaccharides and phospholipids (854 cm^−1^) and the symmetric benzene/pyrrole in-phase and out-of-phase breathing mode of tryptophan and phenylalanine (1004 cm^−1^). Thus, the lack of the *uL11A* gene provided positive feedback response, manifested as enrichment of the metabolome. Additionally, to gain more qualitative insight, principal component analysis (PCA) of selected Raman bands was performed, allowing evaluation of the metabolic cross-correlations between individual yeast mutants (Figure 11).

For the PCA analysis, eight defined peaks in the Raman spectra were considered (Table 2). The analysis of selected peaks showed a cross-correlation between the wild type and the *∆uL11A* deletion strain (Figure 11). In turn, the strains lacking the B isoform or both of them represent two distinct metabolic groups, underscoring fact that the lack of the B or AB isoforms of the uL11 proteins exerts the strongest and diverse effect on yeast metabolism in the qualitative aspect (Figure 11). 

## 4. Discussion

The ribosome, i.e., the mega-Dalton RNA–protein complex, is the central player in translation; importantly, two essential ribosomal catalytic events, namely decoding and peptide-bond-formation, are facilitated by ribosomal RNA (rRNA), with r-proteins playing an indirect role [49]. However, despite the secondary role of r-proteins in the ribosome action, they assist the ribosome in translation from the qualitative and quantitative points of view, contributing significantly to the overall performance of the translational machinery [15,16]. Although they are not directly involved in two crucial ribosomal activities, r-proteins (especially in eukaryotes) serve numerous satellite regulatory functions within the ribosomal particle or allow the ribosome to adjust its translational activity to the cellular needs. A prominent example is the eS6 r-protein, which is implicated in the regulation of so-called TOP mRNA expression and modulation of the translational apparatus in response to various mitogenic stimuli [50]. However, the role of r-proteins seems to be extended beyond translation. It was proposed that r-proteins provide functional heterogeneity to the ribosomes, contributing to “specialized” ribosomes [21]. Additionally, the ribosome can be considered as a “depot”, providing reservoir capacity for r-proteins, which could be released in response to specific cellular cues [22], displaying the so-called extra-ribosomal or moonlighting activity [24]. The additional level of complexity of r-proteins was added by the gene-duplication phenomenon found in almost all organisms; particularly, plants and fungi possess multiple paralogs of r-protein-coding genes [19,20,51]. For example, in plants (*Arabidopsis thaliana*), all r-proteins have at least two paralogs [52]; in *Saccharomyces cerevisiae*, 59 of the 79 r-proteins possess their counterparts [19]. Importantly, 22 out of the 59 duplicated r-proteins in yeast are identical, while 37 differ by one or more amino acid [26,53]. Thus, the maintenance of ∼75% of r-protein paralogs represent a suggestive phenomenon, especially those with an identical amino acid sequence, such as uL11. The genetic links, including genome-wide or single deletion experiments, showed that yeast strains with single gene-deletions of a paralog r-protein frequently showed distinct phenotypes in comparison to strains bearing a paralogous deletion. It was shown that the yeast cell exposed to stress modifies the expression of paralogs by repressing the expression of the major paralog and increasing the number of ribosomes carrying the minor paralog [26]. An additional prominent example was provided by an analysis showing that the bud-tip localized translation of *ASH1* mRNA requires a specific subset of the r-protein [54], supporting the functional model related to “specialized” or “depot” ribosomes. On the other hand, another view, promoting a substantially different idea, is emerging, i.e., the so-called “dosage” model referring to ribosomal availability, which is dependent on the level of r-protein expression, explaining various phenotypes associated with the loss of different r-proteins, especially ribosomopathies [23]. All proposed ideas are not mutually exclusive and point to the multi-level functionality of the r-protein, indicating that every r-protein should be considered as an individual case. In line with this assumption, we focused our attention on the uL11 r-protein, especially in connection with the longevity phenomenon, which has been functionally ascribed to many r-proteins. We used a broad range of analytical methods to provide insight into the role of two paralogs of uL11, i.e., forms A and B. It is thought that perturbations in r-protein expression, which usually lead to disturbances in the translational apparatus, have a positive effect on longevity, especially in the case of the large 60S subunit proteins [14,28,29,30]. However, the mechanisms extending the lifespan in connection with alterations in the translation machinery are not yet fully understood. It has not been resolved whether the extension of the lifespan is directly related to reduction of protein synthesis per se, thus providing a positive energetic pro-longevity effect, or represents extra-ribosomal function of r-proteins. The uL11 r-protein is located at the GAC on the 60S ribosomal subunit, which represents the third active center on the ribosome; the GAC is responsible for interactions with numerous trGTPase and stimulation of their GTPase activity and provides a unidirectional trajectory for the ribosome on mRNA [55]. Thus, the uL11 constitutes an important element supporting GAC in the interplay with trGTPases [33,56], being implicated in all aspects of ribosome activity, including initiation, elongation of the translational cycle, and ribosome biogenesis [36]. Importantly, the lack of the uL11 protein is not deleterious for the ribosome and the cell [36], unlike the other GAC elements, such as the pentameric complex of P-proteins, uL6, or eL40 [30,35,57], which are essential for the ribosome structure and function, and depletion of these elements leads to impairment of the translational machinery. In the study, we used *S. cerevisiae* yeast cells as an experimental model, i.e., single deletion strains *∆uL11A* or *∆uL11B* and double disruptant strain *∆uL11AB* lacking all duplicated genes. Importantly, the uL11 encoded by two genes, for the A and B r-proteins, displays identical amino acid sequences, and our high-throughput RNA sequencing showed a comparable mRNA level for both proteins, raising a question about the role of the A and B isoforms, especially in light of the three proposed “specialized”, “depot”, and “dosage” models. Recently, we showed that the maintenance of two copies of uL6 provides additional supplementation of the r-protein and enables the cell to cope with the high demands for effective ribosome synthesis [30], which is line with the proposed “dosage” model [23]. However, it has also been shown that some paralogs may contribute to stress adaptation, supporting the “specialized” ribosome model [26]. Our initial analyses showed that yeast growth profiling in various environmental conditions did not reveal any significant differences between single deletion strains. However, in the case of *∆uL11B*, sensitivity to hydrogen peroxide was recorded, while the double disruptant mutant displayed sensitivity toward all tested conditions, showing that the yeast mutant strain lacking uL11B or -A/B isoforms has increased sensitivity toward stress conditions, in contrast to the recent observation that perturbations in r-protein expression lead to stress adaptation [26]. The behavior of the *∆uL11AB* strain, displaying a broad spectrum of sensitivity to environmental stimuli, can be explained by the fact that it has significant perturbations in cell cycle (as discussed below), and this defect probably significantly diminishes its adaptation capacity toward various environmental conditions. However, the main question in focus, i.e., the role of the paralogs of uL11 proteins, was analyzed in connection to the longevity issue, which can be analyzed considering the budding lifespan, also called replicative lifespan, or the lifespan expressed in units of time, including the reproductive lifespan and post-reproductive time. Thus, our observation showed significant extension of the budding lifespans of both single mutants, with prominent extension of bud generation in the strain lacking the uL11A r-protein. This observation is supported by reports showing that the lack of rpL22a (eL22A) confers the long-live phenotype, but the lack of rpL22b (eL22B) does not [58]. On the other hand, e.g., in rpL34, loss of either of the paralogs promotes longevity and budding potential [58]. Surprisingly, the reproductive lifespan of the double disruptant was similar to that of the wild-type strain, corroborating the previous observation showing that the rpL22 double paralog deletion is viable, but not long-lived [58]. However, special attention was required by the longevity analysis expressed as a time unit; in the case of the *∆uL11A* mutant, the time was significantly extended, in contrast to *∆uL11B* or *∆uL11AB*, showing that the lack of the uL11A protein exerts the most positive effect on longevity. Previous studies have shown that longevity can be achieved by slowing down the rate of metabolism, for example via deletion of transcription factors [29,38] and a decrease in the growth temperature [59]; especially, perturbations in the protein synthesis rate caused by depletion of r-proteins from the large ribosomal subunit provided the most prominent effect [14,30]. This is in line with the postulated idea that slowing energetically demanding processes, such as translation, may extend the lifespan [9]. However, this was not fully the case in our experimental set-up. The *∆uL11A* long-life mutant displayed an insignificant decrease in the translation rate compared to the wild-type strain, while the *∆uL11B* strain with much lower protein synthesis or especially the *∆uL11AB* strain showed a budding lifespan comparable to that in the isogeneic strain. Interestingly, the 40S/60S ratio in the single deletion mutants was not changed, showing that the depletion of 60S is not critical to extend the budding lifespan and total lifespan. It is worth stressing that not every deletion of r-proteins prolongs the budding lifespan [14]. It was suggested that the expression level of particular paralog genes should be considered [26], as it has been found that some paralog genes for r-proteins are transcribed at a higher level than the others [14]. Interestingly, despite the divergences in the budding lifespan, the uL11 paralogs had comparable mRNA levels, suggesting the observed differences between the A and B isoforms are not related to the dosage of uL11. Additional insight was provided by the metabolic analyses, which showed that the *∆uL11A* long-life mutant displayed the smallest departure from the wild-type strain phenotype; especially, the transcriptomic analysis showed a close cross-correlation of *∆uL11A* with the wild-type strain, with some differences related to the translation-related group of genes and the cell cycle. On the other hand, the *∆uL11B* strain or especially the *∆uL11AB* strain showed the most dramatic changes in the transcriptome, which correlated with the analyzed translational fitness and phenotype analyses, indicating that impairment of the translational machinery does not fully correlate with the longevity phenomenon. Interesting information was provided by Raman spectroscopy, which represents a metabolomic approach on the scale of all chemical compounds in the cell. Once again, the *∆uL11A* long-life mutant showed a chemical pattern correlation with the wild-type and, importantly, enrichment of the chemical composition was observed in some cases, indicating that the lack of the uL11A exerts a positive effect on the chemical metabolome reflected in the extended lifespan. The Raman spectroscopy metabolomic approach is supported by a very recent observation, which showed that yeast strain lacking the rpL22A r-protein, which is long-lived, have lower levels of metabolites associated with 1C metabolism [60], underscoring the fact that the longevity phenomenon is not strictly related to translational slow down. The *∆uL11B* and *∆uL11AB* strains showed a similar metabolomic pattern, departing from the wild type, underscoring once again the fact that greater translational perturbations do not strictly correlate with lifetime extension. Thus, based on our analyses focused on the uL11 r-protein, we claim that the considered energy restriction effect related to depletion of the r-protein and concomitant reduction of translational efficiency does not represent the sole source of the aging phenomenon. 

In our study, we observed an additional interesting phenomenon related to the increase in the yeast cell size, especially in the case of the strain lacking the uL11A or uL11AB r-proteins, referring to the longevity issue. It has been proposed that the budding lifespan is related to the maximum volume that can be achieved by the yeast cell and described as the hypertrophy hypothesis explaining the longevity effect [42]. However, we showed that the *∆uL11A* long-life mutant, having an extended budding lifespan, displayed over a two-fold increase in the maximum volume, compared to the wild type and thus our observation does not support the hypertrophy hypothesis. The double disruptant mutant requires special attention; the strain did not display an extended lifespan, but showed an extraordinary cell size and was accompanied by cell breakdown. The explanation of such behavior is provided by several observations, which showed perturbations in the cell wall metabolism (hypersensitivity to Congo red and calcofluor white), much lower metabolic content determined by Raman spectroscopy, and significantly altered vacuole morphology. Vacuoles are highly dynamic organelles undergoing morphological changes throughout the lifespan and in response to different environmental conditions. The yeast cell typically has several vacuoles, but their number depends on the growth conditions [61]; for example, starvation or osmotic stress results in vacuolar fusion [62,63]. Thus, the observed vacuolar fusion in the *∆uL11AB* strain along with the other observation presented above support the idea that a double disruptant is subjected to severe stress, which exerts deleterious effect on the cell, resulting in the cell breakdown. However, the key data casting more light on the metabolic state of the *∆uL11AB* strain were provided by the transcriptomic analysis. The gene ontology evaluation showed that, in the *∆uL11AB* strain, besides the several pathway perturbations, especially translation and ribosome biogenesis, confirmed by the biochemical analyses (polysome profile or translational fitness), cell cycle disorders were displayed. It was noted that more than 400 genes related to cell cycle progression and mitotic division underwent down regulation. The transcriptomic data were confirmed by flow cytometry, which showed blockage of cell cycle progression in the *∆uL11AB* strain. Additionally, it has also been reported that vacuole integrity represents an important element in yeast cell cycle progression [64]; thus, the observed vacuolar fusion in the double disruptant strain once again supports the involvement of the uL11 cell cycle progression. Recently, it has been suggested that phosphorylation of the uL11 r-protein may be involved in regulation of the cell cycle, implicating this protein in regulation of the translation of specific subsets of mRNAs during mitosis [37]. Our data together with the previous report [37] provide direct evidence that the lack of the uL11 A and B isoforms in the cell perturbs the expression of a large number of genes related to mitotic division, implicating the uL11 protein in regulation of the cell cycle. Thus, we postulate that, besides its important role in the ribosome as a vital element of the GAC, the uL11 displays extra-ribosomal activity in the regulation of the expression of genes involved in the cell cycle. Interestingly, moonlighting activity has also been ascribed to other GAC elements; upon stress conditions, the uL10 (P0) protein can leave the ribosome and act independently, and P1/P2 proteins displayed trans-activation activity [65,66]. Additionally, the eL40 located in the GAC is also ubiquitinated, which affects its fate, and at the same time may influence the formation of the 60S subunit [67]. 

In summary, the integrated biochemical and transcriptomic/metabolic analyses of the yeast mutants lacking the uL11 paralogous r-proteins have provided the following findings. Firstly, the longevity issue, i.e., a phenomenon frequently associated with the role of the translational machinery, analyzed either as replicative or time-scale lifespans, is not fully related to the commonly considered energy restriction effect. Thus, the slow-down of translation (caused by r-protein depletion) does not represent the sole source of the aging phenomenon, and rather subtle changes in the translational machinery determine the cellular proteome, positively affecting the aging process. Secondly, we demonstrated that uL11 is implicated in the cell cycle regulation, thus the proteins might fall into the class of moonlighting r-proteins with extra-ribosomal function. Thus, it can be proposed that the GAC protein elements, including uL11, may fulfill dual functions: support the ribosome in its basic activity and have a regulatory role in cellular metabolism. In line with this assumption, an evolutionary standpoint was proposed that the GAC, being the only ribosomal structure composed of the r-protein, belongs to the last ribosomal element that evolved during ribosome evolution. Consequently, this structure is thought to be responsible for the major breakthrough on the way from RNA to the protein world (considering ribosome molecular evolution), and its appearance together with trGTPases would have dramatically increased the rate and accuracy of protein synthesis, leading to today’s richness of life forms [68,69,70]. Therefore, the late evolutionary addition of GAC r-proteins may represent adaptation of already existing proteins to the ribosomal needs, at the same time maintaining their additional roles. Therefore, we propose that the GAC may constitute a reservoir for proteins with dual function, supporting the “depot model” of the ribosome. 

## Figures and Tables

**Table 1:** Cell cycle analysis in the yeast mutants. The results are shown as means ± SD (n = 4).

Strain	Cell Cycle Phase	
*BY4741*	G1 [%]	17.04 ± 0.39
	G2 [%]	71.94 ± 0.45
	S [%]	11.02 ± 0.65
∆*uL11A*	G1 [%]	28.85 ± 0.76
	G2 [%]	63.05 ± 1.34
	S [%]	8.10 ± 0.76
∆*uL11B*	G1 [%]	19.82 ± 1.88
	G2 [%]	72.09 ± 0.42
	S [%]	8.09 ± 0.98
∆*uL11AB*	G1 [%]	41.88 ± 0.51
	G2 [%]	33.43 ± 0.59
	S [%]	24.67 ± 1.77

**Table 2:** Raman spectroscopy analysis. The individual peak values and their intensity in the analyzed yeast cultures are provided with description of vibrations corresponding to the respective functional groups [44,45,46,47,48]. The data presented are the average values of eight independent repetitions for each object. The significance of the intensity of Raman peaks relative to the yeast being the genetic background (BY4741) was determined using a one-way ANOVA and Tukey’s HSD test procedure at the level of significance: *p* ≤ 0.0005 (***), *p* ≤ 0.005 (**), and *p* ≤ 0.05 (*).

Peak Position [Raman Shift, cm^−1^]	Raman Spectroscopy–Peaks Intensity [a.u.]				Vibrations
	BY4741	∆*uL11A*	∆*uL11B*	∆*uL11AB*	
784	0.027	0.038 ^***^	0.033 ^**^	0.014 ^***^	stretching (O–P–O) RNA
854	0.058	0.065 ^*^	0.060	0.038 ^***^	Polysaccharides, phospholipids
901	0.071	0.079 ^*^	0.079 ^*^	0.050 ^***^	symmetric benzene/pyrrole in-phase and out-of-phase breathing mode of tryptophan and phenylalanine
1004	0.100	0.113 ^**^	0.102	0.056 ^***^	
1100	0.121	0.132	0.133 ^*^	0.089 ^***^	PO_2_ in nucleic acid
1335	0.161	0.169	0.163	0.098 ^***^	C-H deformation vibrations from proteins
1450	0.196	0.205	0.194	0.114 ^***^	C-H deformation vibrations from lipids
1660	0.104	0.105	0.096 ^*^	0.051 ^***^	Amide I: mainly C=O stretching vibrations and contributions of N–H bending vibrations

## Supplementary Materials

The following are available online at https://www.mdpi.com/2073-4409/9/7/1745/s1. Table S1: Cellular component of Gene Ontology (GO) enrichment analysis. GO cellular component includes number of cellular components of upregulated and downregulated genes (Sheet 1). Relative level of mRNA uL11A and uL11B in BY4741 wild-type strain (Sheet 2).
